# Trends in diabetes-related complications in Singapore, 2013–2020: A registry-based study

**DOI:** 10.1371/journal.pone.0275920

**Published:** 2022-10-11

**Authors:** Joshua Kuan Tan, Nur Nasyitah Mohamed Salim, Gek Hsiang Lim, Sing Yi Chia, Julian Thumboo, Yong Mong Bee

**Affiliations:** 1 Health Services Research Unit, Singapore General Hospital, Singapore, Singapore; 2 Department of Endocrinology, Singapore General Hospital, Singapore, Singapore; University of Campania Luigi Vanvitelli: Universita degli Studi della Campania Luigi Vanvitelli, ITALY

## Abstract

**Background:**

Diabetes mellitus (DM) is a growing global health problem. In Singapore, the prevalence of Type 2 DM is rising, but comprehensive information about trends in DM-related complications is lacking.

**Objectives:**

We utilized the Singapore Health Services (SingHealth) diabetes registry (SDR) to assess trends in DM micro and macro-vascular complications at the population level, explore factors influencing these trends.

**Methods:**

We studied trends for ten DM-related complications: ischemic heart disease (IHD), acute myocardial infarction (AMI), peripheral arterial disease (PAD) and strokes, diabetic eye complications, nephropathy, neuropathy, diabetic foot, major and minor lower extremity amputation (LEA). The complications were determined through clinical coding in hospital (inpatient and outpatient) and primary care settings within the SingHealth cluster. We described event rates for the complications in 4 age-bands. Joinpoint regression was used to identify significant changes in trends.

**Results:**

Among 222,705 patients studied between 2013 and 2020. 48.6% were female, 70.7% Chinese, 14.7% Malay and 10.6% Indian with a mean (SD) age varying between 64.6 (12.5) years in 2013 and 65.7 (13.2) years in 2020. We observed an increase in event rates in IHD, PAD, stroke, diabetic eye complications nephropathy, and neuropathy. Joinpoints was observed for IHD and PAD between 2016 to 2018, with subsequent plateauing of event rates. Major and minor LEA event rates decreased through the study period.

**Conclusion:**

We found that DM and its complications represent an important challenge for healthcare in Singapore. Improvements in the trends of DM macrovascular complications were observed. However, trends in DM microvascular complications remain a cause for concern.

## Introduction

Diabetes mellitus (DM) is a growing health problem with a significant global disease burden. In Singapore, the prevalence of diabetes mellitus for citizens and permanent residents aged 18–69 years increased from 8.3% in 2010 to 8.6% in 2017 [1], and the International Diabetes Federation estimates a DM prevalence of 13.7% for the entire adult population of Singapore by 2030 [2]. Singapore is a microcosm of Asia because three major Asian ethnic groups are represented in the city-state: Chinese, Malay and Indian. Also, in the last half century, Singapore has undergone a demographic and epidemiologic transition concomitant with economic development and urbanization, which mirrors ongoing developments in many parts of Asia [3]. Over the last few decades, Singaporeans have been exposed to significant changes in lifestyle, diet, and other environmental influences typical of economic progress and urbanization [4]. The effects of these changes are reflected in the rise in diabetes prevalence. Singapore presages the problems other Asian countries will face as they develop and become increasingly urbanized.

Diabetes imposes a heavy burden on public health and socio-economic development. Uncontrolled diabetes increases the risk of macrovascular and microvascular complications including atherosclerotic cardiovascular disease, ischemic heart disease (IHD), stroke, peripheral arterial disease (PAD), lower extremity amputations (LEA), retinopathy, nephropathy and neuropathy [5]. In Singapore, diabetes accounts for 2.9% of disability-adjusted life years (DALYs) and 4.3% of years lived with disability (YLDs) [6, 7]. In the context of Singapore’s rapidly aging population with steadily increasing life expectancy, morbidity from chronic and non-fatal health problems caused by diabetes continue to present important challenges for the country’s health system [6]. By 2050, annual incident cases of acute myocardial infarction (AMI), stroke and end-stage renal disease (ESRD) amongst residents with diabetes in Singapore are expected to increase by 50% to 16,400 (from 9300 in 2019 for AMI), 12,800 (from 7300 for strokes), and 2700 (from 1700 in 2019 for ESRD) [8]. The increasing burden of disease attributable to DM is expected to drive up healthcare expenditure and lead to greater productivity losses [9]. In response to this, the Singapore government in 2016 declared a “War on Diabetes” (WoD) which comprised a multi-pronged approach involving upstream prevention, early screening and intervention, and better disease control [10]. The early effects of this policy on diabetes prevalence and the disease sequelae have not been comprehensively described.

Knowledge on the trajectory of diabetes prevalence and complications is important to guide effective policy planning and interventions. High-quality, real-world data in the form of disease registries can provide important information for policy making and population health management. Diabetes registries have been used for surveillance, clinical patient management, improving quality of care, research, governance and cost estimation [11]. Diabetes registries are increasingly being utilized for population health management as a component in the chronic care model [12–14].

To address the need for real world data to assess the outcomes of the “War on Diabetes” in Singapore, this study focuses on the Singaporean population within the Singapore Health Services (SingHealth) Regional Health System (RHS) by analyzing data available through the SingHealth Diabetes Registry (SDR). In 2020, the SDR comprised 140,859 patients, representing approximately 20% of DM patients in Singapore [2]. In this paper, we explore using the SDR as a surveillance system to study complications arising from diabetes. Specifically, the aim of this research is to assess changes in diabetic micro and macro-vascular complications, understand the factors influencing these changes and provide insights into the possible population health management policies and interventions.

## Materials and methods

### Population

The multi-institutional SDR has been described previously [15]. Briefly, the SDR is a repository for diabetes-related patient data from across the SingHealth cluster. SingHealth is the largest of 3 public healthcare clusters in Singapore and manages four acute hospitals, five national specialty centres, three community hospitals and a network of nine Polyclinics (SingHealth Polyclinics). The SDR was initiated in 2015 and populated retrospectively and prospectively from SingHealth’s electronic medical records (EMR) and clinical databases to cover the period 2013 to 2020. The registry includes all individuals aged 18 and above with diabetes mellitus, excluding those with pre-diabetes. Cases are annually ascertained using criteria that include diagnosis codes (International Classification of Disease, Nine (ICD-9) and Ten (ICD-10)), prescription records (inpatient or outpatient prescription of glucose lowering drugs or insulin) and laboratory test records (fasting plasma glucose, oral glucose tolerance test, or glycated hemoglobin (HbA1c)). Details of each criterion for identifying diabetes were published earlier [15].

### Outcome ascertainment

We included ten diabetes-related complications in our study. Macrovascular complications include ischemic heart disease (IHD), peripheral arterial disease (PAD) and strokes, whilst microvascular complications include diabetic eye complications (comprising of diabetic retinopathy, maculopathy and other retinal disorders), nephropathy (ascertained from laboratory data) and neuropathy. Our definition of nephropathy was aligned to the Kidney Disease Improving Global Outcomes (KDIGO) 2012 definition of Chronic Kidney Disease (CKD) [16], but included the addition of urine Protein Creatinine Ratio (uPCR) as a criterion because, in our setting, uPCR is used by clinicians to quantify proteinuria [17]. Acute myocardial infarction (AMI), major and minor lower extremity amputation (LEA), and diabetic foot and peripheral angiopathy were also included. We defined major LEAs as any LEA performed at the ankle or above, and minor LEAs as any LEA performed below the ankle. Traumatic amputations were excluded.

The criteria for ascertaining the ten diabetes-related complications included diagnosis codes, surgical codes and laboratory test records and is summarized in Table 1 (detailed description of the criteria is provided in S1 Table). Different healthcare institutions in our healthcare cluster use different standardized diagnosis codes (ICD-10 CM for inpatients, SNOMED-CT for outpatients in tertiary institutions, and SingHealth Polyclinic Working Diagnosis Codes (SHPWDC) for primary care polyclinics). Therefore, we manually defined the relevant diagnostic codes for each setting and outcome. There is no reference standard ICD-10 codes for most diabetes-related complications, and classification and coding practices vary in the literature [18]. We referenced available literature when developing codes for diabetes-related complications [18–21]. As AMI is an outcome of importance, we considered trends in AMI event rates in addition to other ischemic heart disease conditions [22]. As there are no SNOMED-CT reference standard codes for diabetes-related complications, we determined the codes by performing a search for relevant conditions. Surgical codes were referenced from the Ministry of Health Singapore Table of Surgical Procedures [23]. Outcome ascertainment using the coding criterion was facilitated though the SingHealth–Integrated Health Information Systems (IHiS) agency Electronic Health INtelligence System (eHINTS) data repository [15]. In our study, outcomes with multiple recurrences in the calendar year (January 1 to December 31 of that year) were considered a single event.

**Table 1:** Criteria used for outcome ascertainment.

Outcome	Criterion	Healthcare setting
**Macrovascular complications**		
Ischemic heart disease	ICD-10 I20.x, I21.x, I22.x, I24.x, I25.x	Inpatient, outpatient, and primary care.
	SNOMED and SHPWKC codes^a^.	
Acute myocardial infarction	ICD-10 I21.x, I22.x, I25.2	Inpatient
Peripheral arterial disease	ICD-10 I70.2, I73.1, I73.8–73.9.	Inpatient, outpatient, and primary care.
	SNOMED, SHPWKC codes and surgical codes^a^.	
Major and minor lower extremity amputation	Surgical codes (SB010L, SB400T, SB401T, SB707T, SB708T, SB809L, SB829T, SB830T.)	Inpatient
Diabetic foot and peripheral angiopathy	ICD-10 E10.51–10.52, E10.73, E11.51–11.52, E11.73, E13.73, E14.51–14.52, E14.73. SNOMED and SHPWKC codes^a^.	Inpatient, outpatient, and primary care.
Stroke	ICD-10 I60.0–61.9, I62.9, I69.0–69.2, I63.x, I64.x, I69.3 –I69.4, G46.0–46.8.	Inpatient, outpatient, and primary care.
	SNOMED and SHPWKC codes^a^.	
**Microvascular complications**		
Diabetic eye complications	ICD-10 E10.31, E10.39, E11.31–11.39, E14.31–14.39, H330, H332, H334, H342, H348, H350, H352, H353, H354, H358, H431.	Inpatient, outpatient, and primary care.
	SNOMED and SHPWKC codes^a^.	
Nephropathy	eGFR <60mL/min/1.73m^2^ (most recent) and/or uACR ≥ 30mg/g and/or urine protein/creatinine ratio ≥ 0.20 g/g. Laboratory information system data and CKD-EPI formula used to calculate eGFR.	Inpatient, outpatient, and primary care.
Neuropathy	ICD-10 E10.40–10.44, E11.40–11.44, E14.40–14.43, G629. SNOMED and SHPWKC codes^a^.	Inpatient, outpatient, and primary care.

^a^Detailed SNOMED and SHPWKC codes provided in S1 Table.

We evaluated process measures for diabetes care as these might influence the interpretation of complication event rates. Process measures were adapted from the Ministry of Health, Singapore clinical practice guideline [24] and defined as ≥ 2 HbA1c test per year, annual eye screen (diabetic retinal photography and/or visit to ophthalmologists), annual kidney screen (serum creatinine/estimated glomerular filtration rate (eGFR) and/or urine albumin-creatinine ratio (uACR) and/or urine protein-creatinine ratio (uPCR) test), and annual foot screen (diabetic foot screen and/or visits to podiatrists). These measures are similar to process measures specified by the Comprehensive Diabetes Care measures in Health Effectiveness and Information Data Set (HEDIS) used in the United States [25].

### Statistical analysis

SDR data for 2013 to 2020 was used analysis. The SDR contained individuals aged 18 years and above at the time of data capture. The entire registry, comprising of patients with both type 1 and type 2 diabetes, was included in our analysis. Analysis was performed by four age-bands, age-band 1 (18–44 years), age-band 2 (45–64 years), age-band 3 (65–74 years) and age-band 4 (≥ 75 years), to describe the effects of age on the trend estimates. We present age-specific event rates because the underlying population catered to by the SingHealth cluster is dynamic. The SDR cohort experiences the addition (patients newly diagnosed with DM or patients with pre-existing DM but new to the SingHealth system), departure (patients who demise or leave the SingHealth system for treatment in other clusters) and re-entry (patients who leave but re-enter the system at a different time) of patients. When determining event rates, patients present in the registry each year form the denominator and those with the outcome form the numerator. Patients who died were removed from the registry in subsequent years but outcomes during the year of death was included.

Except for AMI, major and minor LEA event rates, all other diabetes-related complications were determined through an expansive clinical coding criteria (S1 Table) and reflect the outcome prevalence in their respective age-bands. AMI event rates were determined from inpatient diagnostic codes only, and thus representative of incidence rates. Major and minor LEA were determined through surgical codes only, and thus representative of incidence rates.

We used Joinpoint regression methodology [26] to analyze the trends in event rates of diabetes-related complications by age-band and to investigate whether there were time points where significant changes in trend occurred. We allowed a maximum of one joinpoint in the models based on the number of observations (8 calendar years) and used a Monte Carlo permutation method for model selection [26].

We performed a sensitivity analysis by excluding events from individuals newly included in the SDR in each study year from the numerator and denominator. We did this to assess the time varying effects of detecting diabetes-related complications in the cohort present in prior years, since there might be a progressive increase in detection of diabetes complications over time through improved diagnostics and screening efforts [27]. We reported the Annual Percent Change (APC) for respective segments as determined by the Joinpoint Regression Program and Average Annual Percent Change (AAPC) for the entire observation period. AAPC is a summary measure of the trend over a pre-specified fixed interval, it describes the average APCs over a period of multiple years. The Joinpoint software calculates the p-value for a two-sided test that the true APC or AAPC is zero using a t-distribution [28].

We performed all analysis using Stata 14.0 (StataCorp. 2015. Stata Statistical Software: Release 14. College Station, TX: StataCorp LP) [29] or Joinpoint Regression Program (Joinpoint Regression Program, Version 4.9.0.1—February 2022; Statistical Methodology and Applications Branch, Surveillance Research Program, National Cancer Institute) [30]. A two-tailed P-value of less than 0.05 was considered statistically significant.

### Ethics approval and informed consent

Ethics approval was obtained from the SingHealth Centralised Institutional Review Board prior to initiating this study (SingHealth CIRB reference number: 2022/2133). As all participant data was de-identified, a waiver for participant consent was also obtained.

## Results

This study included 222,705 patients, with 48.58% females. The population in the registry grew from 92,990 in 2013 to 140,859 in 2020 (Table 2). The SDR population gender and ethnicity structure closely resembled that of the Singapore population [31], comprising of approximately 70% Chinese, 15% Malays and 10% Indians. Almost all patients in the register had type 2 DM (T2DM), a small proportion of patients (<1%) had type 1 or other types (drug-induced, gestational, monogenic and secondary diabetes) of DM. The age structure differed from the Singapore population [31], reflecting the fact that the registry only includes patients with diabetes, of which majority are middle-aged to older adults with T2DM. The mean age varied between 64.6 to 66.0 years during the observation period. There was an increase in mean age of the total population, but the population age structure remained relatively unchanged throughout the observation period (S1 Fig). Minimal variation in the mean age was observed in all age-bands. The majority of subjects were in age-band 2 followed by 3 then 4. The proportion of patients with concomitant hypertension (HTN) and hyperlipidemia (HLD) increased with age. Interestingly, younger age-bands 1 and 2 had higher mean body mass index (BMI), mean HbA1c and mean LDL-cholesterol than age-bands 3 and 4.

**Table 2:** Population structure, demographics, and co-morbidities of the SDR population 2013 to 2020.

		Year							
		2013	2014	2015	2016	2017	2018	2019	2020
Population (n)		92,990	96,846	105,181	111,963	120,221	127,989	134,670	140,859
Percentage of females (%)		50.3	50.0	49.8	49.6	50.2	48.9	48.8	48.3
Ethnicity	Chinese (%)	70.7	70.5	70.6	70.3	71.7	70.1	70.2	69.9
	Malay (%)	14.7	14.9	14.8	14.9	14.4	15.1	14.9	14.8
	Indian (%)	10.6	10.5	10.6	10.7	10.9	10.5	10.5	10.7
	Other (%)	4.1	4.0	4.0	4.0	3.1	4.2	4.4	4.6
Diabetes Type	Type 1 (%)	0.59	0.60	0.64	0.62	0.66	0.73	0.76	0.91
	Type 2 (%)	99.33	99.31	99.27	99.27	99.23	99.15	99.09	98.92
	Others^a^ (%)	0.09	0.09	0.09	0.11	0.11	0.12	0.16	0.17
Population Age structure (years)	Mean (SD)	64.6 (12.5)	64.9 (12.5)	65.1 (12.5)	65.2 (12.5)	66.0 (12.3)	65.4 (12.8)	65.7 (12.8)	65.7 (13.2)
	Median age	65	65	65	66	66	66	66	67
	IQR	57–74	57–74	57–74	57–74	58–74	58–74	58–74	58–74
	Min, Max	18, 105	18, 104	18, 105	18, 106	18, 105	18, 118	18, 113	18, 108
**By Age-band**									
Age-band 1 (18–44 years)	Count (n)	5,254	5,366	5,776	6,237	5,894	7,538	8,037	8,759
	Percent of total population (%)	5.7	5.5	5.5	5.6	4.9	5.9	6.0	6.2
	Mean age (SD)	37.1 (6.5)	37.1 (6.5)	37.1 (6.4)	36.9 (6.6)	37 (6.5)	36.7 (6.4)	36.6 (6.3)	36.7 (6.2)
	**Co-morbidities** ^b^									
	DM only (%)	3.5	3.3	3.6	3.5	6.2	6.0	6.7	6.0
	DM + HTN only (%)	4.2	3.4	4.2	3.7	4.6	4.7	4.8	4.4
	DM + HLD only (%)	26.2	28.6	27.8	31.0	32.5	35.1	34.8	34.8
	DM + HTN + HLD (%)	66.0	64.7	64.5	61.8	56.7	54.2	53.7	54.8
	Mean BMI (Kg/m^2^) (SD)	25.7 (5.4)	30.0 (6.7)	30.1 (6.6)	30.1 (6.8)	30.2 (6.8)	30.6 (6.9)	30.6 (6.9)	30.8 (7.1)
	Mean HbA1c (%) (SD)	8.2 (2.1)	8.1 (2.1)	8.2 (2.1)	8.1 (2.0)	8.0 (2.0)	7.8 (2.0)	7.7 (2.0)	7.8 (2.1)
	Mean LDL-C (mmol/L) (SD)	2.80 (0.96)	2.84 (0.94)	2.79 (0.99)	2.66 (0.98)	2.56 (0.91)	2.64 (0.95)	2.64 (0.93)	2.61 (0.96)
Age-band 2 (45–64 years)	Count (n)	40,298	40,936	43,582	45,799	46,303	50,271	51,255	51,956
	Percent of total population (%)	43.3	42.3	41.4	40.9	38.5	39.3	38.1	36.9
	Mean age (SD)	56.8 (5.2)	56.8 (5.2)	56.9 (5.1)	57 (5.2)	57.2 (5.1)	57 (5.2)	57.1 (5.2)	57.2 (5.2)
	**Co-morbidities** ^b^									
	DM only (%)	0.9	0.9	0.9	0.8	1.3	1.6	1.5	1.2
	DM + HTN only (%)	3.5	3.1	3.1	2.1	3.1	3.3	3.3	2.7
	DM + HLD only (%)	13.1	13.4	13.2	13.5	15.3	16.4	16.9	15.6
	DM + HTN + HLD (%)	82.5	82.5	82.8	83.6	80.4	78.7	78.3	80.5
	Mean BMI (Kg/m^2^) (SD)	27.4 (5.1)	27.4 (5.0)	27.5 (5.1)	27.4 (5.2)	27.4 (5.3)	27.5 (5.3)	27.6 (5.4)	27.7 (5.5)
	Mean HbA1c (%) (SD)	7.6 (1.6)	7.4 (1.6)	7.6 (1.6)	7.6 (1.6)	7.6 (1.6)	7.4 (1.6)	7.4 (1.6)	7.5 (1.6)
	Mean LDL-C (mmol/L) (SD)	2.46 (0.84)	2.51 (0.85)	2.48 (0.83)	2.39 (0.81)	2.24 (0.79)	2.29 (0.84)	2.30 (0.84)	2.25 (0.84)
Age-band 3 (65–74 years)	Count (n)	26,587	28,154	31,006	33,221	38,044	39,538	42,578	45,705
	Percent of total population (%)	28.6	29.1	29.5	29.7	31.6	30.9	31.6	32.4
	Mean age (SD)	69.2 (3)	69.1 (2.9)	69.1 (2.8)	69.0 (2.8)	69.2 (2.8)	69.2 (2.8)	69.3 (2.8)	69.4 (2.8)
	**Co-morbidities** ^b^									
	DM only (%)	0.4	0.5	0.4	0.4	0.6	0.7	0.7	0.5
	DM + HTN only (%)	3.3	2.9	3.0	1.8	3.2	3.1	3.1	2.2
	DM + HLD only (%)	5.1	5.4	5.5	5.2	7.2	7.9	8.2	7.0
	DM + HTN + HLD (%)	91.2	91.2	91.2	92.6	89.0	88.3	88.0	90.2
	Mean BMI (Kg/m^2^) (SD)	25.9 (4.6)	25.9 (4.5)	26.0 (4.6)	25.8 (4.5)	25.8 (4.6)	25.9 (4.7)	25.9 (4.7)	26.0 (4.9)
	Mean HbA1c (%) (SD)	7.2 (1.3)	7.0 (1.3)	7.2 (1.3)	7.1 (1.2)	7.2 (1.2)	7.0 (1.3)	7.1 (1.3)	7.2 (1.3)
	Mean LDL-C (mmol/L) (SD)	2.27 (0.73)	2.32 (0.74)	2.29 (0.73)	2.20 (0.71)	2.07 (0.68)	2.10 (0.73)	2.10 (0.73)	2.05 (0.73)
Age-band 4 (≥ 75 years)	Count (n)	20,851	22,390	24,817	26,706	29,980	30,642	32,800	34,061
	Percent of total population (%)	22.4	23.1	23.6	23.9	24.9	23.9	24.4	24.2
	Mean age (SD)	80.8 (4.8)	80.8 (4.8)	80.9 (4.9)	81.0 (4.9)	81.2 (4.9)	81.3 (5.1)	81.5 (5.1)	81.6 (5.2)
	**Co-morbidities** ^b^									
	DM only (%)	0.3	0.3	0.3	0.2	0.4	0.3	0.4	0.2
	DM + HTN only (%)	3.9	3.5	3.5	1.9	3.7	3.2	3.4	2.2
	DM + HLD only (%)	3.6	3.5	3.5	2.6	4.3	4.4	4.9	3.5
	DM + HTN + HLD (%)	92.1	92.7	92.7	95.4	91.6	92.0	91.3	94.1
	Mean BMI (Kg/m^2^) (SD)	24.8 (4.6)	24.8 (4.4)	24.8 (4.5)	24.5 (4.4)	24.5 (4.5)	24.6 (4.8)	24.5 (4.6)	24.4 (4.6)
	Mean HbA1c (%) (SD)	6.9 (1.2)	6.8 (1.2)	6.9 (1.2)	7.0 (1.2)	7.1 (1.2)	6.9 (1.2)	6.9 (1.3)	7.1 (1.3)
	Mean LDL-C (mmol/L) (SD)	2.21 (0.72)	2.25 (0.74)	2.21 (0.71)	2.13 (0.69)	2.03 (0.70)	2.02 (0.70)	2.02 (0.71)	1.97 (0.72)

^a^ Other diabetes type include drug-induced, gestational, monogenic and secondary diabetes.

^b^ Co-morbidities was tabulated based on available data and does not include all patients in the registry.

We confirmed that the SDR is a dynamic cohort (S2 Fig). We observed that the population distribution and mean age within each age age-band remained stable, indicating that direct comparison within age-band and across years can yield meaningful conclusions about age specific event rates.

### Trends in macrovascular event rates

**Fig 1:**
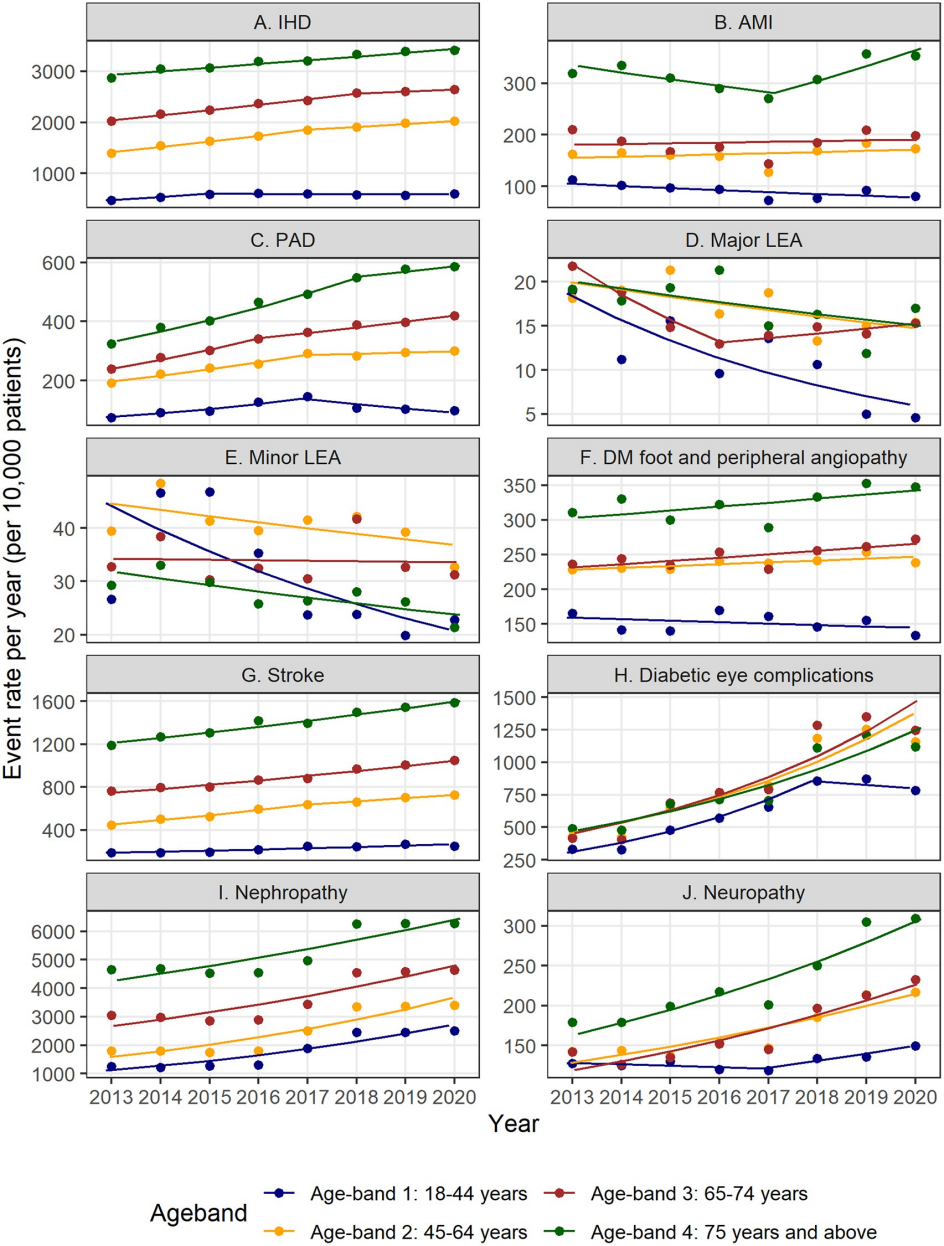
Trends in diabetes complications rates by age-bands. Event rates for (A) ischemic heart disease, (B) acute myocardial infraction, (C) peripheral arterial disease, (D) major LEA, (E) minor LEA, (F) diabetic foot and peripheral angiopathy, (G) stroke, (H) diabetic eye complications, (I) nephropathy and (J) neuropathy. Dots are observed event rates. Solid lines are modeled event rates from the Joinpoint regression analysis.

From 2013 to 2020, there was an observed rise in event rates for most macrovascular complications except major and minor LEAs (Fig 1, Table 3, S2 and S3 Tables). An increased prevalence of IHD, PAD and stroke can be inferred from the data. For IHD, a statistically significant increase in event rates for all age-bands, as determined by the AAPCs, was observed during the observation period. Joinpoints were identified for IHD between 2015 and 2018 in age-bands 1 to 3, with smaller APC values in the subsequent segments (although not statistically significant for age-bands 1 and 3) suggesting a possible reduction in the rate of increase in prevalence of IHD in recent years.

**Table 3:** Joinpoint analysis of trends in event rates of diabetes-related complications by age-band.

Co-morbidity / Age band	Event rate (per 10,000)			Time period 1		Time period 2	
	2013	2020	AAPC (95% CI)	Year	APC (95% CI)	Year	APC (95% CI)
**Ischemic Heart Disease**							
18–44 years	475.8	598.2	3.1 (0.4, 5.8)*	2013–2015	12.5 (-1.8, 28.9)	2015–2020	-0.5 (-2.8, 1.9)
45–64 years	1397.3	2023.3	5.2 (4.2, 6.3)*	2013–2017	6.9 (4.7, 9.1)***	2017–2020	3.1 (0.4, 5.8)*
65–74 years	2028.1	2643.9	3.7 (2.8, 4.6)*	2013–2018	4.6 (3.4, 5.8)***	2018–2020	1.5 (-2.5, 5.6)
≥ 75 years	2868.9	3407.1	2.3 (1.7, 2.9)*	2013–2020	2.3 (1.7, 2.9)***		
**Acute myocardial infraction**							
18–44 years	112.3	81.1	-4.1 (-7.6, -0.5)*	2013–2020	-4.1 (-7.6, -0.5)*		
45–64 years	162.8	173.2	1.4 (-2.4, 5.4)	2013–2020	1.4 (-2.4, 5.4)		
65–74 years	210.3	198.5	0.8 (-3.9, 5.7)	2013–2020	0.8 (-3.9, 5.7)		
≥ 75 years	319.4	352.9	1.4 (-2.8, 5.7)	2013–2017	-4.2 (-12, 4.3)	2017–2020	9.4 (-2.1, 22.2)
**Peripheral Arterial Disease**							
18–44 years	74.2	99.3	3.1 (-0.7, 7.0)	2013–2017	16.2 (7.6, 25.4)**	2017–2020	-12.1 (-20.3, -3.1)*
45–64 years	191.8	301.4	6.1 (4.3, 8.1)*	2013–2017	9.9 (5.9, 14)**	2017–2020	1.4 (-3.3, 6.2)
65–74 years	238.8	420.3	8.2 (6.5, 10.1)*	2013–2016	12.5 (6.6, 18.8)**	2016–2020	5.2 (2.7, 7.7)**
≥ 75 years	323.7	585.1	8.4 (6.0, 10.9)*	2013–2018	10.6 (7.1, 14.3)**	2018–2020	3.1 (-6.9, 14.2)
**Major LEA**							
18–44 years	19.0	4.6	-14.9 (-23.8, -4.9)*	2013–2020	-14.9 (-23.8, -4.9)*		
45–64 years	18.1	15.4	-4.2 (-8.5, 0.3)	2013–2020	-4.2 (-8.5, 0.3)		
65–74 years	21.8	15.3	-5.0 (-8.0, -1.8)*	2013–2016	-15.6 (-23.4, -6.9)*	2016–2020	3.9 (-1.9, 9.9)
≥ 75 years	19.2	17.0	-4.0 (-9.6, 1.9)	2013–2020	-4.0 (-9.6, 1.9)		
**Minor LEA**							
18–44 years	26.7	22.8	-10.2 (-18.6, -0.9)*	2013–2020	-10.2 (-18.6, -0.9)*		
45–64 years	39.5	32.7	-2.7 (-6.1, 0.8)	2013–2020	-2.7 (-6.1, 0.8)		
65–74 years	32.7	31.3	-0.3 (-5.2, 5.0)	2013–2020	-0.3 (-5.2, 5.0)		
≥ 75 years	29.3	21.4	-4.1 (-7.1, -1.0)*	2013–2020	-4.1 (-7.1, -1.0)*		
**Diabetic foot and peripheral angiopathy**							
18–44 years	165.6	133.6	-1.4 (-4.6, 2.0)	2013–2020	-1.4 (-4.6, 2.0)		
45–64 years	228.5	238.7	1.1 (0.2, 2.1)*	2013–2020	1.1 (0.2, 2.1)*		
65–74 years	236.6	272.2	2.0 (0.3, 3.7)*	2013–2020	2.0 (0.3, 3.7)*		
≥ 75 years	310.8	347.3	1.8 (-0.5, 4.2)	2013–2020	1.8 (-0.5, 4.2)		
**Stroke**							
18–44 years	188.4	246.6	5.2 (2.6, 8.0)*	2013–2020	5.2 (2.6, 8.0)**		
45–64 years	443.2	725.0	7.1 (5.4, 8.9)*	2013–2017	9.2 (5.6, 12.9)**	2017–2020	4.5 (0.2, 8.9)*
65–74 years	762.0	1046.9	5.0 (4.2, 5.8)*	2013–2020	5.0 (4.2, 5.8)***		
≥ 75 years	1189.9	1586.9	4.0 (3.2, 4.8)*	2013–2020	4.0 (3.2, 4.8)***		
**Diabetic eye complications**							
18–44 years	331.2	784.3	15.0 (8.9, 21.4)*	2013–2018	23.1 (13.1, 34)**	2018–2020	-3.1 (-22.6, 21.2)
45–64 years	429.1	1158.7	17.1 (10.3, 24.4)*	2013–2020	17.1 (10.3, 24.4)**		
65–74 years	417.1	1246.9	18.2 (9.8, 27.3)*	2013–2020	18.2 (9.8, 27.3)**		
≥ 75 years	488.2	1118.9	14.9 (8.6, 21.5)*	2013–2020	14.9 (8.6, 21.5)**		
**Nephropathy**							
18–44 years	1250.5	2510.6	13.5 (7.8, 19.5)*	2013–2020	13.5 (7.8, 19.5)**		
45–64 years	1798.1	3396.3	12.7 (7.0, 18.8)*	2013–2020	12.7 (7.0, 18.8)**		
65–74 years	3050.4	4636.7	8.7 (4.3, 13.3)*	2013–2020	8.7 (4.3, 13.3)**		
≥ 75 years	4663.1	6268.8	6.0 (2.8, 9.3)*	2013–2020	6.0 (2.8, 9.3)**		
**Neuropathy**							
18–44 years	127.5	149.6	2.1 (-0.5, 4.8)	2013–2017	-1.4 (-6.6, 4.1)	2017–2020	7.1 (0.1, 14.6)*
45–64 years	142.7	216.9	7.6 (4.2, 11.2)*	2013–2020	7.6 (4.2, 11.2)**		
65–74 years	142.2	232.4	9.6 (5.7, 13.7)*	2013–2020	9.6 (5.7, 13.7)**		
≥ 75 years	179.4	309.4	9.4 (6.1, 12.8)*	2013–2020	9.4 (6.1, 12.8)***		

^†^ Joinpoint regression software provided indication whether AAPC p-value is significant at p<0.05, no detailed p-values were provided by the software.

^‡^ Joinpoint regression software provides detailed p-values for APC.

* P < 0.05

** P < 0.01

*** P < 0.005

A negative and statistically significant AAPC for AMI event rates in age-band 1 was observed, indicating declining AMI event rates amongst younger patients. As assessed by their respective AAPCs, we did not observe any statistically significant trend in AMI event rates for age-bands 2 to 4.

Positive and statistically significant AAPCs for PAD in age-bands 2 to 4 was observed. Joinpoint regression demonstrated a decline in event rates for age-band 1 and lower rate of increase of event rates for age-bands 2 to 4, suggesting some degree of plateauing. Declines in event rates for both major and minor LEAs was observed, with statistically significant declines seen in age-band 1 and 3 for major LEA, and in age-bands 1 and 4 for minor LEA. Event rates for diabetic foot and peripheral angiopathy showed minimal increase during the observation period. Statistically significant increases in stroke event rates were observed across all age-bands. A Joinpoint was identified in age-band 2, with reduction in rate of increase in age-band 2 in between 2017–2020 as compared to prior years.

### Trends in microvascular event rates

Marked increases in event rates for microvascular complications was observed (Fig 1, S2 and S3 Tables). We observed an increase in diabetic eye complications event rates, as determined by the corresponding AAPCs and a statistically significant year-on-year increase of at least 15% in all age-bands. A Joinpoint was identified for diabetic eye complications in age-band 1, but the APC for the later segment was not statistically significant. Large increase in event rates were also observed for nephropathy and neuropathy with disproportionately larger increases in event rates for younger age-bands. A Joinpoint was identified for neuropathy in age-band 1, the positive and statistically significant APC in the later segment indicates an increase in neuropathy event rates between 2017–2020. Process measures for diabetes care of patients were comparable to similar measures across the United States and Japan [32–34]. In this study, the process measures exhibited some variation but did not demonstrate marked increase over the observation period (S4 Table).

### Sensitivity analysis

Sensitivity analysis excluding patients newly included in the SDR in each year (S5 Table) yielded similar trends in rates of diabetes-related complications. We observed marginally larger AAPCs values for diabetic eye complications, nephropathy and neuropathy suggesting progressive increase in detection of diabetes-related complications over time through improved diagnostics and screening efforts on the cohort that was present in prior years.

## Discussion

In our analysis of the SingHealth Diabetes Registry, we identified substantial increases in event rates for seven out of ten major diabetes-related macro- and microvascular complications including ischemic heart disease, peripheral arterial disease, stroke, diabetic eye complications, nephropathy, and neuropathy between 2013 and 2020. Between 2016 to 2018, we observed plateauing of event rates for ischemic heart disease and peripheral arterial disease in age-bands 2 to 4. Event rates of diabetic foot and peripheral angiopathy remained stable with minimal increase, concurrent decline in major and minor LEA event rates was noted throughout the observation period. Overall, these findings indicate that whilst there is continued increase in the prevalence of some diabetes-related complications, this trend is moderating and may show signs of reversing in some complications.

Our findings provide important insights into the epidemiology of diabetes-related complications in Singapore and indicate that T2DM continue to represent an important challenge for healthcare in Singapore. Congruent with international studies, continued efforts to reduce the chronic, non-fatal health problems caused by diabetes are required [35]. We observed that although diabetic patients from age-band 1 amounted to approximately 5% of the population in the registry, they are an important population group. In our study, this group had higher mean BMI, HbA1c and LDL-C, and are therefore likely to be at greater risk for atherogenic cardiovascular disease and diabetic complications in the future [36]. Compared to age-bands 1 and 2, age-bands 3 and 4 had the lower mean HbA1c and LDL-C values with the smaller standard deviations suggesting better control and less variation in glycemic and lipid control.

Improvements in mean HbA1c and LDL-C were observed for all age-bands from 2013 to 2019. However, the work of Lind et al. suggests that this would take a longer period to result in a reduction in event-rates for diabetes-related complications [37]. However, plateauing of event rates of IHD and PAD in recent years could be a harbinger of a future reduction in event rates. Interestingly, a marginal increase in mean HbA1c was noted in 2020 for all age-bands, this might be due to widespread healthcare system disruption due to the COVID-19 pandemic. Future analysis of the SDR could explore the effects of pandemic related healthcare disruption on the management of diabetes and associated complications.

### Trends in diabetes-related macrovascular complications

The increasing trend in event rates for diabetes-related macrovascular complications in the SDR cohort is concerning. Data from large population-based studies in Europe and North America suggest that the risk of CVD in patients with diabetes has been declining since the 1990s [18]. In Hong Kong, the event rates for coronary heart disease, stroke, and heart failure based on hospital principal diagnosis codes at discharge and amongst diabetic patients have been decreasing from 2001 to 2016 [27]. In South Korea, rates of hospitalization due to cardiovascular complications (ischemic heart disease, ischemic stroke, and myocardial infarction) among diabetic patients decreased from 2006 to 2015; however, increase in rates of heart failure, and PAD was observed [38]. Comparable population-level statistics spanning the whole spectrum of healthcare is limited and little is known about the rates of diabetic complications beyond the inpatient setting.

However, the increasing trend in macrovascular complications observed in our study is not unexpected, as Tan et al. projected an increase in incident cases in AMI, stroke and end stage renal disease in Singapore until 2050 [8]. In addition, our observation of statistically significant increases in stroke event rates across all age-bands is consistent with the findings of Feng et al. who observed an increase in 10-year stroke risk in the SDR between 2013 and 2019 [39]. The increasing trend in macrovascular complications might be due to evolving demographic structure in Singapore with increase in obesity prevalence, particularly amongst ethnic Indians and Malays [40], and earlier onset of DM which increases the risk of developing sequelae [8]. Similar increase in prevalence of coronary artery disease, cerebrovascular disease and peripheral artery disease have been reported in amongst T2DM patients ≥ 65 years throughout public hospitals in Thailand between 2010 and 2015 [41]. In the United States, incidence of hospitalization due to AMI, stroke, LEA initially decreased from 1990 to 2010 [42] but increased by more than 25% during the 5 years from 2010 to 2015 for adults aged 18 to 64 years [43]. Published data on trends of diabetes-related macrovascular complications from 2015 onwards is limited.

### Trends in diabetes-related microvascular complications

Statistically significant decline in major and minor LEA event rates was observed in age-band 1 and borderline significant declines in major LEA event rates was seen in age-bands 2 and 3. The observed trend in SDR contrasts with national data which showed stable trends in major and toe/ray LEA rates for diabetic patients with no statistically significant decline between 2008 and 2017 [44]. Declining trends in LEA has been observed in many countries in Australia, Europe, Hong Kong and Thailand [18, 27, 41, 45]. Explaining this reduction in LEA event rates is not straightforward. LEAs are an important indicator of the success of preventive care, targeted glycemic control, cardiovascular disease risk factor management, and screening and treatment of people at high risk of foot complications [18]. We observed decreasing event rates for both major and minor LEAs suggesting that improvements in major LEAs did not occur because minor LEAs were performed in the clinical setting to prevent major LEAs. Statistically significant increase in PAD rates was observed, and since PAD was also ascertained with surgical codes for revascularization procedures, it may be possible that early revascularization could have resulted in lower major and minor LEA event rates. Additionally, efforts by a National Diabetic Foot Workgroup could have contributed to the reduction on LEAs through early multidisciplinary care for patients with diabetic foot in polyclinics and public hospitals [46]. Further research to investigate the pathways to amputation and the role of preventive care measures (e.g. diabetic foot screening), pharmacologic strategies (e.g. stringent glycemic and lipid control) and intervention therapies (e.g. early revascularization procedures) in the local context are needed to better understand the reasons for the improvement in major and minor LEA rates observed [44].

Statistically significant increase in event rates for microvascular complications were observed in the SDR, especially in diabetic eye complications, nephropathy and neuropathy. This increase in event rates cannot be explained by increase in eye, foot or kidney screening as the rates for these process measures remained fairly constant throughout the observation period.

In our study, the diabetic eye complications assessed consists of several conditions, including diabetic retinopathy, diabetic maculopathy and other diabetes associated retinal conditions. Event rates for diabetic eye complications appeared to sharply increase during the observation period. The progressive implementation of electronic health records (EHR) system at the Singapore National Eye Centre (SNEC), the specialty centre for eye care within SingHealth, may explain these trends. Earlier in the observation period, SNEC had yet to fully implement an EHR system to record outpatient clinical diagnoses. Thus, diabetic eye complication data would not be captured in the SingHealth–IHiS eHINTS data repository. Presently, SNEC has a fully operational EHR system, therefore future analysis might provide more accurate insight into diabetic eye complication rates. Despite incomplete data early in the observation period, other studies confirm that diabetic eye complications are a concern for ophthalmologist and public health planners in Singapore. Locally, population based studies estimated the prevalence of DR to be between 28.2% [47] and 33.9% [48], higher than other Asian countries but comparable to the Western world. A recent meta-analysis determined that the prevalence of DR, vision-threatening DR and clinically significant macular edema amongst diabetic patients to be 19.20% (CI: 14.16, 25.50), 5.54% (CI: 4.53, 6.76), 3.23% (CI: 2.26, 4.59) respectively in the Western pacific region (which includes Singapore), with the prevalence of DR expected to increase into the future [49]. It has been postulated that projected increase in DR could be due to the “legacy effect” in diabetes, where epigenomic alterations from poor glycemic control early in the disease course result in sustained vascular dysfunction despite improvements in glycemic control [50, 51]. In the context of our findings, it is imperative that efforts be concentrated towards glycemic control in all diabetic patients to mitigate potential “legacy effects” and improve eye screening rates to detect and treat diabetic eye complications at an early stage.

The trends in event rate for nephropathy amongst diabetic patients is worrying. Our results suggest an increase in prevalence of nephropathy in all age-bands with a corresponding prevalence of 25.1%, 34.0%, 46.4% and 62.7% in age-bands 1 to 4 in 2020 (respectively). Studies on the epidemiology of CKD secondary to T2DM in Singapore are limited. A cross-sectional study at a different healthcare cluster in Singapore demonstrated the prevalence of diabetes-related CKD to be 53% [52]. According to the United States Renal Data System (USRDS), Singapore ranks second, behind South Korea, in average yearly increase in incidence of treated end stage renal disease (ESRD) attributed to diabetes [53]. Steep increases in incidence of diabetes-associated ESRD have been reported for Taiwan, Malaysia, Qatar and United States [18, 53]. It is likely that most cases of nephropathy are secondary to DM, but it is unclear what proportion of nephropathy can be attributable to other causes such as hypertension and glomerulonephritides, and what are the drivers for the high prevalence of CKD in T2DM patients in Singapore when compared to other Asian countries [54]. Nonetheless, the increasing event rates for nephropathy amongst diabetic patients have important public health implications due to increase in healthcare utilization. Lim et al. using the same definition of CKD, found that in Singapore, diabetic patients with CKD utilized more healthcare resources (outpatient visits and inpatient hospitalizations) than those without CKD, and the annual mean cost for diabetic patients with CKD was 2.2 times higher than those without CKD [55]. Additionally, the mean annual cost of CKD increases with the severity of CKD stage. Tan et al. project that by 2050, 70% of incident cases of ESRD in Singapore will have concomitant DM [8]. Concerted efforts are underway to prevent the occurrence of DM induced nephropathy and retard the progression to ESRD. Notably, such efforts include the Holistic Approach in Lowering and Tracking CKD (HALT-CKD) programme and the Diabetes Study in Nephropathy and other Microvascular Complications, both initiated in 2017. The former is a primary care intervention which systematically identify, track all CKD patients and help control disease risk factors, while the latter is a large genomic study to improve the understand of diabetic kidney disease and reduce its prevalence in Singapore [56].

### Implications

Our study reveals some improvement and plateauing of IHD and PAD event rates, but the continual rise in prevalence of microvascular complications is worrisome. The projected increase in diabetes prevalence [2] and the uptrend in diabetes-related complications presented in this study represent important challenges for Singapore’s healthcare system. Diabetic complications which occur in middle age results in more years of healthy life lost due to disability (YLD) than deaths [35], resulting in poorer quality of life and increase healthcare utilization. From a policy perspective, steps must be taken to control the rise in the prevalence of diabetes-related complications and to prevent further strain to the healthcare system. It has been suggested that the nexus of care must shift from the hospital to the community and primary care must be the foundation for a sustainable health system [57]. The comprehensive outcome data and process measures obtained from the SDR has allowed us to generate valuable insights on population health and highlight areas for clinical improvement. Future uses of the SDR may include program evaluations for value driven care, generating important case-mix information and facilitate performance benchmarking as Singapore moves towards capitation based healthcare financing in Singapore [58, 59].

### Strengths

In this study, we conducted a thorough longitudinal analysis of diabetes-related macro- and microvascular complications amongst a multi-ethnic cohort in Singapore. To our knowledge, this is the largest study of diabetic patients in Singapore to date. Since there is currently no widely agreed upon standard for defining diabetes complications from clinical and administrative data [60], we utilized a detailed case ascertainment methodology spanning primary, hospital outpatient and inpatient care. This represents a novel approach to comprehensively determine diabetes complications rate from across a wide range of clinical settings. The outcomes ascertained and the event rates determined are therefore more likely to reflect the prevalence and burden of disease in the population than outcomes determined from hospitalization admissions alone.

### Limitations

Limitations of our study include the dynamic nature of the SDR population, especially patients who leave the registry each year. This is affected by the fluid nature of the healthcare landscape in Singapore where patients are free to choose their healthcare providers and health data from patients who seek care in other public healthcare clusters or in the private sector is currently not available for research by SingHealth. The lack of data about diabetes complications diagnosed in other healthcare clusters or in the private sector may affect our results and confuse the overview of diabetes complications in the study population. However, our sensitivity analysis confirms that our findings remain robust for the patients who remain in the prior years’ cohort. Another limitation was the inability to distinguish between incident and prevalent cases for most of the outcomes evaluated. Perhaps future studies could attempt to triangulate incident events using hospital discharge diagnoses and other algorithms. Further, explaining the trends in macro- and microvascular event rates observed is difficult because of the myriad of potential factors in outcome ascertainment, data acquisition, preventive care, patient characteristics and behaviors [43]. Lastly, given that SingHealth adopts a multi-pronged and multifactorial approach to the management of diabetes and it’s complications, future research using the SDR could explore the efficacy the multifactorial approach to diabetes management [61].

## Conclusions

We analyzed data from a large-scale diabetes registry and identified substantial increase in event rates for seven out of ten major diabetes-related macro- and microvascular complications. Event rates for ischemic heart disease and peripheral arterial disease demonstrate some plateauing but event rates for microvascular complications continue to increase. We demonstrated that the SDR provides comprehensive and precise data reflecting the burden of disease in the SingHealth cluster and can be used for planning and evaluating healthcare interventions that span the continuum of care.

## Supporting information

S1 Fig Population age structure box plot from 2013 to 2020. (DOCX)

S2 Fig Population movement in the SDR cohort. (DOCX)

S1 Table Detailed criteria used for outcome ascertainment. (DOCX)

S2 Table Event counts and event rates for all outcomes 2013–2016. (DOCX)

S3 Table Event counts and event rates for all outcomes 2017–2020. (DOCX)

S4 Table Percent of patients with type 1 and 2 DM satisfying process measure. (DOCX)

S5 Table Sensitivity analysis of trends in event rates of diabetes-related complications by age-band, excluding patients newly included to the SDR in each study year. (DOCX)
