# Supplementary figures and images for: Trends in diabetes-related complications in Singapore, 2013–2020: A registry-based study
Source: PLoS One. 2022 Oct 11;17(10):e0275920. doi: 10.1371/journal.pone.0275920 (PMC9553054; doi:10.1371/journal.pone.0275920)

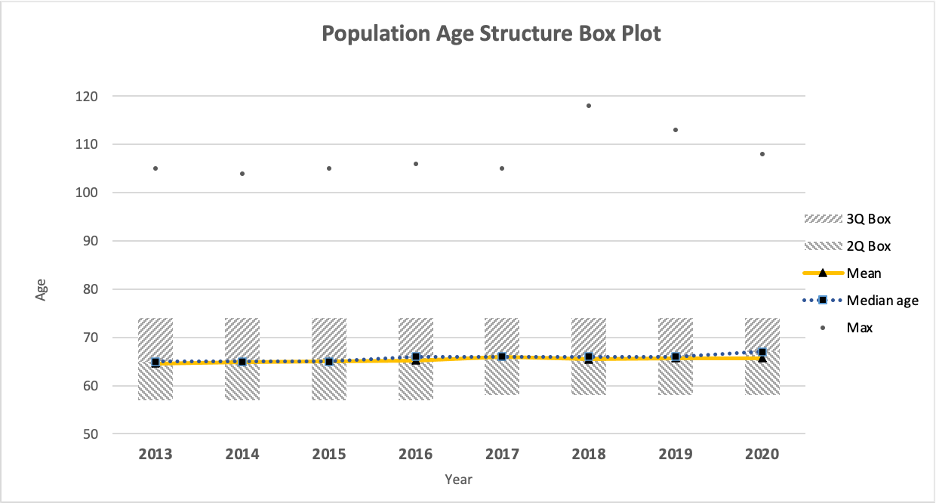


S1Fig. Population Age Structure Box Plot from 2013 to 2020.

Supplement: S1 Fig — (DOCX) [file pone.0275920.s001.docx]

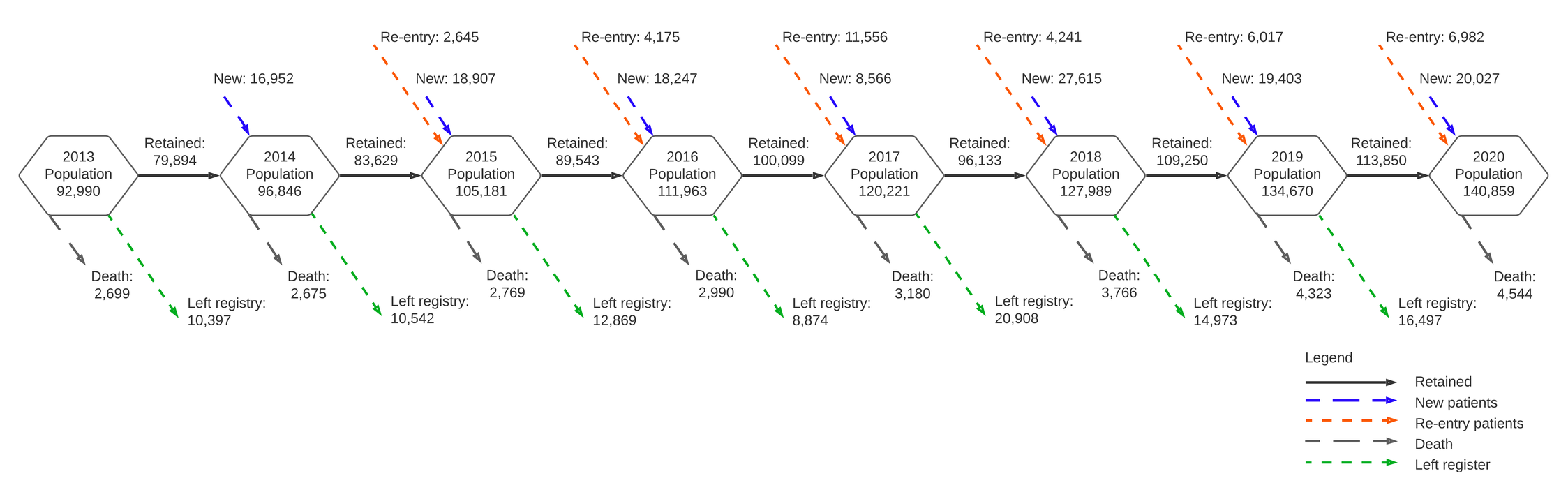


S2 Fig. Population movement in the SDR cohort.

Supplement: S2 Fig — (DOCX) [file pone.0275920.s002.docx]
