# Supplementary material for: Trends in diabetes-related complications in Singapore, 2013–2020: A registry-based study
Source: PLoS One. 2022 Oct 11;17(10):e0275920. doi: 10.1371/journal.pone.0275920 (PMC9553054; doi:10.1371/journal.pone.0275920)
Supplement: S1 Table — (DOCX) [file pone.0275920.s003.docx]

**S1 Table. Detailed criteria used for outcome ascertainment.**

| Outcome | Criterion | | | Healthcare setting |
| --- | --- | --- | --- | --- |
|  | ICD-10 codes | SNOMED codes | SHPWKC or Surgical codes |  |
| Macrovascular complications | | | | |
| Ischemic heart disease | ICD-10 I20.x, I21.x, I22.x, I24.x, I25.x | 89331010, 299761018, 299762013, 299782012, 299783019, 299784013, 350346019, 459859010, 1040100013, 1051100013, 1210398011, 1210399015, 1210505015, 2534663012, 2534664018, 2534667013, 2534671011, 2534672016, 2535853014, 2535854015, 2535923015, 2536394018, 2536395017, 2536397013, 2536398015, 2537479013, 2537480011, 2842265012, 2951007019, 2951008012, 2951009016, 2951010014, 2986368010, 2986970011, 3289323011 | 042A (Chronic ischemic heart disease), 042B (Acute ischemic heart disease), | Inpatient, outpatient and primary care. |
| Acute myocardial infarction | ICD-10 I21.x, I22.x, I 25.2 | - | - | Inpatient |
| Peripheral arterial disease | ICD-10 I70.2, I73.1, I73.8-73.9 | 18413012, 23786012, 46785011, 87530017, 105536013, 205224017, 300607015, 350531010, 350534019, 350535018, 350536017, 350543011, 350544017, 350545016, 350546015, 350547012, 350548019, 357894012, 357896014, 357897017, 357898010, 411512011, 443199013, 451376017, 456609017, 456610010, 456617013, 458506014, 458507017, 473473019, 484263016, 1232467011, 1233009011, 1779239017, 1779317016, 1787049010, 1787050010, 1787052019, 2157354010, 2162392019, 2693498010, 2693575017, 2693576016, 2773672016, 2921338010, 2923092011, 2923094012, 2923309015, 2923379016, 2955895013, 2956118016, 2983747016, 2983776018, 2985567012, 3040857018, 3289284017 | 047B (PVD), surgical codes (SD720A (Artery, Stenosis / Occlusion, Percutaneous Transluminal Angioplasty (PTA), Difficult (Eg Subintimal PTA, Below Knee PTA)), SD728A (Artery, Various Lesions, Endovascular Stent Placement), SD713A (Artery, Bypass, Above/Below-Knee With Vein), SD717A (Artery, Femoral Artery Angioplasty), SD721A (Artery, Stenosis / Occlusion, Percutaneous Transluminal Angioplasty (Pta), Simple), SD714A (Artery, Bypass, Distal Leg/Pedal With Vein), SD719A (Artery, Stenosis / Occlusion, Percutaneous Atherectomy (Mechanical Or Laser))) | Inpatient, outpatient and primary care. |
| Major or minor lower extremity amputation | - | - | Mapped from surgical codes (SB010L, SB400T, SB401T, SB707T, SB708T, SB809L, SB829T, SB830T.) |  |
| Diabetic foot and peripheral angiopathy | ICD-10 E10.51-10.52, E10.73, E11.51-11.52, E11.73, E13.73, E14.51-14.52, E14.73 | 286569015, 286570019, 300505017, 300506016, 308360012, 309178017, 357897017, 357898010, 357902016, 417658010, 451404016, 451405015, 498448012, 512081010, 1209795012, 1488395018, 1488396017, 1778315019, 1781954013, 2532976019, 2532977011, 2533104014, 2621545012, 2621551019, 2621797015, 2621798013, 2987211019, 3040857018 | 018K (DM Foot) | Inpatient, outpatient and primary care. |
| Stroke | ICD-10 I60.0-61.9, I62.9, I69.0-69.2, I63.x, I64.x, I69.3 – I69.4, G46.0-46.8 | 3421016, 13756016, 36011016, 39067013, 39068015, 42129011, 49074018, 79273012, 96391014, 123408011, 124627016, 124628014, 124629018, 134436018, 158109012, 158113017, 158114011, 158118014, 178528011, 251701010, 300271012, 300272017, 300277011, 300279014, 300321011, 300322016, 300365015, 300366019, 300369014, 300408017, 345635016, 345636015, 345637012, 345638019, 345640012, 345641011, 345642016, 345646018, 345647010, 345649013, 345651012, 345652017, 345653010, 345656019, 345659014, 345667018, 345671015, 345671015, 345675012, 345676013, 345677016, 345682011, 345694019, 345696017, 405377010, 405379013, 409859018, 409860011, 412270012, 412997011, 419212013, 419213015, 444898010, 450609012, 451133011, 451134017, 451371010, 475553012, 481028017, 481580017, 496232015, 502878012, 503469016, 1209750017, 1209751018, 1212072018, 1216125018, 1217312010, 1217630014, 1218800017, 1229580019, 2474651019, 2474986019, 2475187018, 2476091017, 2535208016, 2644233012, 2644234018, 2674093010, 2674127019, 2675250011, 2694009014, 2695743015, 2770034014, 2772089016, 2818561018, 2819959010, 2819960017, 2901452016, 2901453014, 2914970017, 2916313015, 2920376012, 2966556014, 2966565019, 2966596011, 2966602014, 2966650013, 2967471017, 2967506011, 2967537011, 2967598015, 2967601013, 2967609010, 2967628015, 2967650019, 2967701015, 2981993016, 2984150019, 2984188017, 2984239014, 2986393012, 2986825016, 2986886017, 2987976012, 3023367013, 3023374015, 3037871010, | 046A (Stroke (Haemorrhage)), 046C (Stroke (Ischaemic/infarct)), 046B (Stroke (Not specified) | Inpatient, outpatient and primary care. |
| Microvascular complications | | | | |
| Ophthalmopathy | ICD-10 E10.31, E10.39, E11.31-11.39, E14.31-14.39, H330, H332, H334, H342, H348, H350, H352, H353, H354, H358, H431 | 9093013, 33037011, 42066015, 42768011, 52406015, 70200018, 89378011, 93460014, 98476015, 158593013, 251652015, 297723010, 297745011, 297802010, 297803017, 297806013, 297807016, 347642015, 347645018, 347650012, 347651011, 347657010, 347660015, 347720013, 347721012, 368990015, 368991016, 456707011, 456712012, 456713019, 456714013, 456715014, 456716010, 456717018, 484848010, 486663012, 486665017, 493192015, 1228202019, 1228203012, 1230610015, 1231068014, 1484867016, 1488434016, 1773466014, 1774748012, 1774750016, 1775724010, 1775725011, 1779161016, 1779166014, 1783717018, 1783897011, 1784103011, 1785151013, 1785152018, 1785155016, 1785158019, 1785161018, 1785332013, 2164671014, 2164673012, 2551706016, 2579342012, 2579343019, 2579521014, 2618238018, 2618241010, 2620592018, 2620593011, 2621399015, 2621472018, 2621576018, 2642672017, 2643005014, 2921114017, 2967698011, 2967760017, 2967787018, 2989730012, 2990445013, 3004798011, 3013241019, 3303450013, | 018Q (DM Retinopathy) | Inpatient, outpatient and primary care. |
| Nephropathy | Criteria: eGFR <60mL/min/1.73m^2^ (most recent) and/or urine albumin-creatinine ratio (uACR) ≥ 30mg/g and/or urine protein/creatinine ratio ≥ 0.20 g/g. Laboratory information system data and CKD-EPI formula used to calculate eGFR. | | |  |
| Neuropathy | ICD-10 E10.40-10.44, E11.40-11.44, E14.40-14.43, G629 | 299011, 8504012, 23368011, 34691019, 59668014, 65526011, 65642012, 70659018, 82373015, 84344019, 94571018, 94572013, 100945019, 100951012, 131983013, 135747015, 141590010, 158470017, 158471018, 170583016, 194672015, 195776019, 206412019, 255456017, 255459012, 255462010, 255465012, 297550019, 297551015, 297552010, 323630017, 345486016, 345487013, 345488015, 345489011, 345490019, 345491015, 345492010, 345524019, 369417015, 413629013, 414606010, 417464011, 473862018, 475273015, 477766010, 478654013, 480783010, 480785015, 480788018, 502316014, 1230890017, 1480220018, 1483041015, 1493575013, 1777429019, 1778546016, 1786560016, 1786593013, 1786596017, 1786598016, 2645902011, 2667589016, 2667594016, 2694146010, 2870944013, 2920418018, 2967619016, 2967625011, 2967635011, 2967939019, 3005273015, 3286275018, 3286275018, 3297353013 | 018P (DM Neuropathy) | Inpatient, outpatient and primary care. |
