# Supplementary material for: Trends in diabetes-related complications in Singapore, 2013–2020: A registry-based study
Source: PLoS One. 2022 Oct 11;17(10):e0275920. doi: 10.1371/journal.pone.0275920 (PMC9553054; doi:10.1371/journal.pone.0275920)
Supplement: S2 Table — (DOCX) [file pone.0275920.s004.docx]

**S2 Table. Event counts and event rates for all outcomes 2013 – 2016.**

|  | **Event counts** | **Event rate (per 10,000) [95% C.I.]** |  | **Event counts** | **Event rate (per 10,000) [95% C.I.]** |  | **Event counts** | **Event rate (per 10,000) [95% C.I.]** |  | **Event counts** | **Event rate (per 10,000) [95% C.I.]** |
| --- | --- | --- | --- | --- | --- | --- | --- | --- | --- | --- | --- |
| **Year** | **2013** | |  | **2014** | |  | **2015** | |  | **2016** | |
| **IHD** | | | | | | | | | | | |
| 18-44 years | 250 | 475.8 (418.3, 533.4) |  | 286 | 533.0 (472.9, 593.1) |  | 344 | 595.6 (534.5, 656.6) |  | 380 | 609.3 (549.9, 668.6) |
| 45-64 years | 5631 | 1397.3 (1363.5, 1431.2) |  | 6326 | 1545.3 (1510.3, 1580.3) |  | 7113 | 1632.1 (1597.4, 1666.8) |  | 7912 | 1727.6 (1692.9, 1762.2) |
| 65-74 years | 5392 | 2028.1 (1979.7, 2076.4) |  | 6101 | 2167.0 (2118.9, 2215.1) |  | 6952 | 2242.2 (2195.7, 2288.6) |  | 7863 | 2366.9 (2321.2, 2412.6) |
| ≥ 75 years | 5982 | 2868.9 (2807.5, 2930.3) |  | 6834 | 3052.3 (2991.9, 3112.6) |  | 7625 | 3072.5 (3015.1, 3129.9) |  | 8533 | 3195.2 (3139.2, 3251.1) |
| **AMI** | | | | | | | | | | | |
| 18-44 years | 59 | 112.3 (83.8, 140.8) |  | 55 | 102.5 (75.5, 129.4) |  | 56 | 97.0 (71.7, 122.2) |  | 59 | 94.6 (70.6, 118.6) |
| 45-64 years | 656 | 162.8 (150.4, 175.1) |  | 675 | 164.9 (152.6, 177.2) |  | 698 | 160.2 (148.4, 171.9) |  | 726 | 158.5 (147.1, 170.0) |
| 65-74 years | 559 | 210.3 (193.0, 227.5) |  | 528 | 187.5 (171.7, 203.4) |  | 518 | 167.1 (152.8, 181.3) |  | 586 | 176.4 (162.2, 190.5) |
| ≥ 75 years | 666 | 319.4 (295.5, 343.3) |  | 748 | 334.1 (310.5, 357.6) |  | 769 | 309.9 (288.3, 331.4) |  | 773 | 289.4 (269.3, 309.6) |
| **PAD** | | | | | | | | | | | |
| 18-44 years | 39 | 74.2 (51.0, 97.4) |  | 49 | 91.3 (65.9, 116.8) |  | 56 | 97.0 (71.7, 122.2) |  | 80 | 128.3 (100.3, 156.2) |
| 45-64 years | 773 | 191.8 (178.4, 205.2) |  | 908 | 221.8 (207.5, 236.1) |  | 1056 | 242.3 (227.9, 256.7) |  | 1174 | 256.3 (241.9, 270.8) |
| 65-74 years | 635 | 238.8 (220.5, 257.2) |  | 785 | 278.8 (259.6, 298.1) |  | 935 | 301.6 (282.5, 320.6) |  | 1135 | 341.7 (322.1, 361.2) |
| ≥ 75 years | 675 | 323.7 (299.7, 347.7) |  | 853 | 381.0 (355.9, 406.1) |  | 997 | 401.7 (377.3, 426.2) |  | 1242 | 465.1 (439.8, 490.3) |
| **Major LEA** | | | | | | | | | | | |
| 18-44 years | 10 | 19.0 (7.2, 30.8) |  | 6 | 11.2 (2.2, 20.1) |  | 9 | 15.6 (5.4, 25.8) |  | 6 | 9.6 (1.9, 17.3) |
| 45-64 years | 73 | 18.1 (14.0, 22.3) |  | 78 | 19.1 (14.8, 23.3) |  | 93 | 21.3 (17.0, 25.7) |  | 75 | 16.4 (12.7, 20.1) |
| 65-74 years | 58 | 21.8 (16.2, 27.4) |  | 53 | 18.8 (13.8, 23.9) |  | 46 | 14.8 (10.6, 19.1) |  | 43 | 12.9 (9.1, 16.8) |
| ≥ 75 years | 40 | 19.2 (13.2, 25.1) |  | 40 | 17.9 (12.3, 23.4) |  | 48 | 19.3 (13.9, 24.8) |  | 57 | 21.3 (15.8, 26.9) |
| **Minor LEA** | | | | | | | | | | | |
| 18-44 years | 14 | 26.6 (12.7, 40.6) |  | 25 | 46.6 (28.4, 64.8) |  | 27 | 46.7 (29.2, 64.3) |  | 22 | 35.3 (20.6, 50.0) |
| 45-64 years | 159 | 39.5 (33.3, 45.6) |  | 198 | 48.4 (41.6, 55.1) |  | 180 | 41.3 (35.3, 47.3) |  | 181 | 39.5 (33.8, 45.3) |
| 65-74 years | 87 | 32.7 (25.9, 39.6) |  | 108 | 38.4 (31.1, 45.6) |  | 94 | 30.3 (24.2, 36.4) |  | 108 | 32.5 (26.4, 38.6) |
| ≥ 75 years | 61 | 29.3 (21.9, 36.6) |  | 74 | 33.1 (25.5, 40.6) |  | 74 | 29.8 (23, 36.6) |  | 69 | 25.8 (19.7, 31.9) |
| **DM foot and peripheral angiopathy** | | | | | | | | | | | |
| 18-44 years | 87 | 165.6 (131.1, 200.1) |  | 76 | 141.6 (110.0, 173.3) |  | 81 | 140.2 (109.9, 170.6) |  | 106 | 170.0 (137.9, 202.0) |
| 45-64 years | 921 | 228.5 (214.0, 243.1) |  | 943 | 230.4 (215.8, 244.9) |  | 999 | 229.2 (215.2, 243.3) |  | 1101 | 240.4 (226.4, 254.4) |
| 65-74 years | 629 | 236.6 (218.3, 254.9) |  | 687 | 244.0 (226.0, 262.0) |  | 727 | 234.5 (217.6, 251.3) |  | 843 | 253.8 (236.8, 270.7) |
| ≥ 75 years | 648 | 310.8 (287.2, 334.3) |  | 739 | 330.1 (306.7, 353.5) |  | 744 | 299.8 (278.6, 321.0) |  | 860 | 322.0 (300.9, 343.2) |
| **Stroke** | | | | | | | | | | | |
| 18-44 years | 99 | 188.4 (151.7, 225.2) |  | 102 | 190.1 (153.5, 226.6) |  | 110 | 190.4 (155.2, 225.7) |  | 136 | 218.1 (181.8, 254.3) |
| 45-64 years | 1786 | 443.2 (423.1, 463.3) |  | 2058 | 502.7 (481.6, 523.9) |  | 2281 | 523.4 (502.5, 544.3) |  | 2717 | 593.2 (571.6, 614.9) |
| 65-74 years | 2026 | 762.0 (730.1, 793.9) |  | 2240 | 795.6 (764.0, 827.2) |  | 2477 | 798.9 (768.7, 829.1) |  | 2873 | 864.8 (834.6, 895) |
| ≥ 75 years | 2481 | 1189.9 (1145.9, 1233.8) |  | 2838 | 1267.5 (1223.9, 1311.1) |  | 3245 | 1307.6 (1265.6, 1349.5) |  | 3787 | 1418 (1376.2, 1459.9) |
| **Diabetic eye complications** | | | | | | | | | | | |
| 18-44 years | 174 | 331.2 (282.8, 379.6) |  | 175 | 326.1 (278.6, 373.7) |  | 276 | 477.8 (422.8, 532.9) |  | 356 | 570.8 (513.2, 628.4) |
| 45-64 years | 1729 | 429.1 (409.3, 448.8) |  | 1729 | 422.4 (402.9, 441.9) |  | 2829 | 649.1 (626.0, 672.3) |  | 3409 | 744.3 (720.3, 768.4) |
| 65-74 years | 1109 | 417.1 (393.1, 441.2) |  | 1136 | 403.5 (380.5, 426.5) |  | 2133 | 687.9 (659.8, 716.1) |  | 2552 | 768.2 (739.6, 796.8) |
| ≥ 75 years | 1018 | 488.2 (459.0, 517.5) |  | 1069 | 477.4 (449.5, 505.4) |  | 1682 | 677.8 (646.5, 709.0) |  | 1910 | 715.2 (684.3, 746.1) |
| **Nephropathy** | | | | | | | | | | | |
| 18-44 years | 657 | 1250.5 (1161.0, 1339.9) |  | 657 | 1224.4 (1136.7, 1312.1) |  | 740 | 1281.2 (1195.0, 1367.4) |  | 812 | 1301.9 (1218.4, 1385.4) |
| 45-64 years | 7246 | 1798.1 (1760.6, 1835.6) |  | 7342 | 1793.5 (1756.4, 1830.7) |  | 7626 | 1749.8 (1714.1, 1785.5) |  | 8225 | 1795.9 (1760.7, 1831.0) |
| 65-74 years | 8110 | 3050.4 (2995, 3105.7) |  | 8401 | 2983.9 (2930.5, 3037.4) |  | 8878 | 2863.3 (2813.0, 2913.6) |  | 9627 | 2897.9 (2849.1, 2946.7) |
| ≥ 75 years | 9723 | 4663.1 (4595.4, 4730.8) |  | 10490 | 4685.1 (4619.8, 4750.5) |  | 11267 | 4540.0 (4478.1, 4602.0) |  | 12144 | 4547.3 (4487.6, 4607.0) |
| **Neuropathy** | | | | | | | | | | | |
| 18-44 years | 67 | 127.5 (97.2, 157.9) |  | 67 | 124.9 (95.1, 154.6) |  | 75 | 129.8 (100.6, 159.0) |  | 75 | 120.3 (93.2, 147.3) |
| 45-64 years | 575 | 142.7 (131.1, 154.3) |  | 590 | 144.1 (132.6, 155.7) |  | 596 | 136.8 (125.9, 147.7) |  | 704 | 153.7 (142.4, 165.0) |
| 65-74 years | 378 | 142.2 (127.9, 156.4) |  | 355 | 126.1 (113.1, 139.1) |  | 419 | 135.1 (122.3, 148.0) |  | 506 | 152.3 (139.1, 165.5) |
| ≥ 75 years | 374 | 179.4 (161.4, 197.4) |  | 401 | 179.1 (161.7, 196.5) |  | 495 | 199.5 (182.1, 216.9) |  | 581 | 217.6 (200.1, 235.1) |
