# Supplementary material for: Trends in diabetes-related complications in Singapore, 2013–2020: A registry-based study
Source: PLoS One. 2022 Oct 11;17(10):e0275920. doi: 10.1371/journal.pone.0275920 (PMC9553054; doi:10.1371/journal.pone.0275920)
Supplement: S3 Table — (DOCX) [file pone.0275920.s005.docx]

**S3 Table. Event counts and event rates for all outcomes 2017 – 2020.**

|  |  | **Event counts** | **Event rate (per 10,000) [95% C.I.]** |  | **Event counts** | **Event rate (per 10,000) [95% C.I.]** |  | **Event counts** | **Event rate (per 10,000) [95% C.I.]** |  | **Event counts** | **Event rate (per 10,000) [95% C.I.]** |
| --- | --- | --- | --- | --- | --- | --- | --- | --- | --- | --- | --- | --- |
| **Year** |  | **2017** | |  | **2018** | |  | **2019** | |  | **2020** | |
| **Ischemic heart disease** | | | | | | | | | | | | |
| 18-44 years |  | 356 | 604.0 (543.2, 664.8) |  | 441 | 585.0 (532.1, 638.0) |  | 462 | 574.8 (523.9, 625.7) |  | 524 | 598.2 (548.6, 647.9) |
| 45-64 years |  | 8559 | 1848.5 (1813.1, 1883.8) |  | 9588 | 1907.3 (1872.9, 1941.6) |  | 10162 | 1982.6 (1948.1, 2017.2) |  | 10512 | 2023.3 (1988.7, 2057.8) |
| 65-74 years |  | 9239 | 2428.5 (2385.4, 2471.6) |  | 10171 | 2572.5 (2529.4, 2615.6) |  | 11097 | 2606.3 (2564.6, 2648.0) |  | 12084 | 2643.9 (2603.5, 2684.3) |
| ≥ 75 years |  | 9604 | 3203.5 (3150.6, 3256.3) |  | 10217 | 3334.3 (3281.5, 3387.1) |  | 11116 | 3389.0 (3337.8, 3440.3) |  | 11605 | 3407.1 (3356.8, 3457.5) |
| **Acute myocardial infarction** | | | | | | | | | | | | |
| 18-44 years |  | 43 | 73.0 (51.2, 94.7) |  | 58 | 76.9 (57.2, 96.7) |  | 74 | 92.1 (71.2, 113.0) |  | 71 | 81.1 (62.3, 99.8) |
| 45-64 years |  | 590 | 127.4 (117.2, 137.6) |  | 852 | 169.5 (158.2, 180.8) |  | 940 | 183.4 (171.8, 195.0) |  | 900 | 173.2 (162.0, 184.4) |
| 65-74 years |  | 549 | 144.3 (132.3, 156.3) |  | 732 | 185.1 (171.9, 198.4) |  | 889 | 208.8 (195.2, 222.4) |  | 907 | 198.4 (185.7, 211.2) |
| ≥ 75 years |  | 810 | 270.2 (251.8, 288.5) |  | 943 | 307.7 (288.4, 327.1) |  | 1171 | 357.0 (336.9, 377.1) |  | 1202 | 352.9 (333.3, 372.5) |
| **Peripheral arterial disease** | | | | | | | | | | | | |
| 18-44 years |  | 86 | 145.9 (115.3, 176.5) |  | 81 | 107.5 (84.2, 130.7) |  | 84 | 104.5 (82.3, 126.8) |  | 87 | 99.3 (78.6, 120.1) |
| 45-64 years |  | 1355 | 292.6 (277.3, 308.0) |  | 1428 | 284.1 (269.5, 298.6) |  | 1511 | 294.8 (280.2, 309.4) |  | 1566 | 301.4 (286.7, 316.1) |
| 65-74 years |  | 1380 | 362.7 (343.9, 381.5) |  | 1536 | 388.5 (369.4, 407.5) |  | 1690 | 396.9 (378.4, 415.5) |  | 1921 | 420.3 (401.9, 438.7) |
| ≥ 75 years |  | 1478 | 493.0 (468.5, 517.5) |  | 1683 | 549.2 (523.7, 574.8) |  | 1893 | 577.1 (551.9, 602.4) |  | 1993 | 585.1 (560.2, 610.1) |
| **Major LEA** | | | | | | | | | | | | |
| 18-44 years |  | 8 | 13.6 (4.2, 23.0) |  | 8 | 10.6 (3.3, 18.0) |  | 4 | 5.0 (0.1, 9.9) |  | 4 | 4.6 (0.1, 9.0) |
| 45-64 years |  | 87 | 18.8 (14.8, 22.7) |  | 67 | 13.3 (10.1, 16.5) |  | 77 | 15.0 (11.7, 18.4) |  | 80 | 15.4 (12.0, 18.8) |
| 65-74 years |  | 53 | 13.9 (10.2, 17.7) |  | 59 | 14.9 (11.1, 18.7) |  | 60 | 14.1 (10.5, 17.7) |  | 70 | 15.3 (11.7, 18.9) |
| ≥ 75 years |  | 45 | 15 (10.6, 19.4) |  | 50 | 16.3 (11.8, 20.8) |  | 39 | 11.9 (8.2, 15.6) |  | 58 | 17.0 (12.6, 21.4) |
| **Minor LEA** | | | | | | | | | | | | |
| 18-44 years |  | 14 | 23.8 (11.3, 36.2) |  | 18 | 23.9 (12.9, 34.9) |  | 16 | 19.9 (10.2, 29.7) |  | 20 | 22.8 (12.8, 32.8) |
| 45-64 years |  | 192 | 41.5 (35.6, 47.3) |  | 212 | 42.2 (36.5, 47.8) |  | 201 | 39.2 (33.8, 44.6) |  | 170 | 32.7 (27.8, 37.6) |
| 65-74 years |  | 116 | 30.5 (25.0, 36.0) |  | 165 | 41.7 (35.4, 48.1) |  | 139 | 32.6 (27.2, 38.1) |  | 143 | 31.3 (26.2, 36.4) |
| ≥ 75 years |  | 79 | 26.4 (20.5, 32.2) |  | 86 | 28.1 (22.1, 34.0) |  | 86 | 26.2 (20.7, 31.8) |  | 73 | 21.4 (16.5, 26.3) |
| **DM foot and peripheral angiopathy** | | | | | | | | | | | | |
| 18-44 years |  | 95 | 161.2 (129.0, 193.3) |  | 110 | 145.9 (118.9, 173.0) |  | 125 | 155.5 (128.5, 182.6) |  | 117 | 133.6 (109.5, 157.6) |
| 45-64 years |  | 1102 | 238.0 (224.1, 251.9) |  | 1214 | 241.5 (228.1, 254.9) |  | 1301 | 253.8 (240.2, 267.4) |  | 1240 | 238.7 (225.5, 251.8) |
| 65-74 years |  | 872 | 229.2 (214.2, 244.2) |  | 1012 | 256.0 (240.4, 271.5) |  | 1113 | 261.4 (246.2, 276.6) |  | 1244 | 272.2 (257.3, 287.1) |
| ≥ 75 years |  | 867 | 289.2 (270.2, 308.2) |  | 1020 | 332.9 (312.8, 353) |  | 1157 | 352.7 (332.8, 372.7) |  | 1183 | 347.3 (327.9, 366.8) |
| **Stroke** | | | | | | | | | | | | |
| 18-44 years |  | 147 | 249.4 (209.6, 289.2) |  | 183 | 242.8 (208, 277.5) |  | 216 | 268.8 (233.4, 304.1) |  | 216 | 246.6 (214.1, 279.1) |
| 45-64 years |  | 2944 | 635.8 (613.6, 658.0) |  | 3328 | 662.0 (640.3, 683.7) |  | 3601 | 702.6 (680.4, 724.7) |  | 3767 | 725.0 (702.7, 747.3) |
| 65-74 years |  | 3357 | 882.4 (853.9, 910.9) |  | 3832 | 969.2 (940.0, 998.4) |  | 4287 | 1006.9 (978.3, 1035.4) |  | 4785 | 1046.9 (1018.9, 1075) |
| ≥ 75 years |  | 4180 | 1394.3 (1355.1, 1433.5) |  | 4590 | 1497.9 (1458, 1537.9) |  | 5060 | 1542.7 (1503.6, 1581.8) |  | 5405 | 1586.9 (1548.1, 1625.7) |
| **Diabetic eye complications** | | | | | | | | | | | | |
| 18-44 years |  | 387 | 656.6 (593.4, 719.8) |  | 645 | 855.7 (792.5, 918.8) |  | 700 | 871.0 (809.3, 932.6) |  | 687 | 784.3 (728.0, 840.6) |
| 45-64 years |  | 3781 | 816.6 (791.6, 841.5) |  | 5958 | 1185.2 (1156.9, 1213.4) |  | 6425 | 1253.5 (1224.9, 1282.2) |  | 6020 | 1158.7 (1131.2, 1186.2) |
| 65-74 years |  | 3006 | 790.1 (763, 817.2) |  | 5087 | 1286.6 (1253.6, 1319.6) |  | 5746 | 1349.5 (1317.1, 1382) |  | 5699 | 1246.9 (1216.6, 1277.2) |
| ≥ 75 years |  | 2112 | 704.5 (675.5, 733.4) |  | 3407 | 1111.9 (1076.7, 1147.1) |  | 3963 | 1208.2 (1173.0, 1243.5) |  | 3811 | 1118.9 (1085.4, 1152.4) |
| **Nephropathy** | | | | | | | | | | | | |
| 18-44 years |  | 1109 | 1881.6 (1781.8, 1981.4) |  | 1851 | 2455.6 (2358.4, 2552.7) |  | 1974 | 2456.1 (2362.0, 2550.3) |  | 2199 | 2510.6 (2419.7, 2601.4) |
| 45-64 years |  | 11636 | 2513.0 (2473.5, 2552.5) |  | 16891 | 3360.0 (3318.7, 3401.3) |  | 17239 | 3363.4 (3322.5, 3404.3) |  | 17646 | 3396.3 (3355.6, 3437.1) |
| 65-74 years |  | 13067 | 3434.7 (3387.0, 3482.4) |  | 18001 | 4552.8 (4503.7, 4601.9) |  | 19548 | 4591.1 (4543.8, 4638.4) |  | 21192 | 4636.7 (4591.0, 4682.4) |
| ≥ 75 years |  | 14907 | 4972.3 (4915.7, 5028.9) |  | 19164 | 6254.2 (6200.0, 6308.4) |  | 20583 | 6275.3 (6223.0, 6327.6) |  | 21352 | 6268.8 (6217.4, 6320.1) |
| **Neuropathy** | | | | | | | | | | | | |
| 18-44 years |  | 70 | 118.8 (91.1, 146.4) |  | 101 | 134 (108.0, 159.9) |  | 109 | 135.6 (110.3, 160.9) |  | 131 | 149.6 (124.1, 175) |
| 45-64 years |  | 680 | 146.9 (135.9, 157.8) |  | 933 | 185.6 (173.8, 197.4) |  | 1097 | 214.0 (201.5, 226.6) |  | 1127 | 216.9 (204.4, 229.4) |
| 65-74 years |  | 552 | 145.1 (133.1, 157.1) |  | 778 | 196.8 (183.1, 210.5) |  | 907 | 213.0 (199.3, 226.7) |  | 1062 | 232.4 (218.5, 246.2) |
