# Supplementary material for: Trends in diabetes-related complications in Singapore, 2013–2020: A registry-based study
Source: PLoS One. 2022 Oct 11;17(10):e0275920. doi: 10.1371/journal.pone.0275920 (PMC9553054; doi:10.1371/journal.pone.0275920)
Supplement: S4 Table — (DOCX) [file pone.0275920.s006.docx]

**S4 Table. Percent of patients with type 1 and 2 DM satisfying process measure.**

|  | Process measures | Year | | | | | | | |
| --- | --- | --- | --- | --- | --- | --- | --- | --- | --- |
|  |  | 2013 | 2014 | 2015 | 2016 | 2017 | 2018 | 2019 | 2020 |
| Percent of population satisfying process measure (%) | ≥ 2 HbA1c tests per year | 56.9 | 55.1 | 54.5 | 53.8 | 53.1 | 56.0 | 55.9 | 57.4 |
|  | Annual eye screening^1^ | 43.1 | 44.9 | 45.5 | 46.2 | 46.9 | 44.0 | 44.1 | 42.6 |
|  | Annual kidney screening^2^ | 66.9 | 63.9 | 63.3 | 62.4 | 61.0 | 68.4 | 69.5 | 72.5 |
|  | Annual diabetic foot screening^3^ | 39.0 | 37.3 | 37.8 | 39.3 | 36.9 | 37.1 | 35.0 | 33.4 |

^1^Diabetic retinal photography (DRP) and/or visit to ophthalmologists.

^2^Serum creatinine/estimated glomerular filtration rate (eGFR) and/or urine albumin-creatinine ratio (uACR) and/or urine protein-creatinine ratio (uPCR) test performed.

^3^Diabetic foot screen (DFS) and/or visits to podiatrists.
