# Supplementary material for: Trends in diabetes-related complications in Singapore, 2013–2020: A registry-based study
Source: PLoS One. 2022 Oct 11;17(10):e0275920. doi: 10.1371/journal.pone.0275920 (PMC9553054; doi:10.1371/journal.pone.0275920)
Supplement: S5 Table — (DOCX) [file pone.0275920.s007.docx]

**S5 Table. Sensitivity analysis of trends in event rates of diabetes-related complications by age-band, excluding patients newly included to the SDR in each study year.**

| **Co-morbidity / Age band** | | **Event rate (per 10,000)** | | |  | **Time period 1** | |  | **Time period 2** | |
| --- | --- | --- | --- | --- | --- | --- | --- | --- | --- | --- |
|  |  | **2014** | **2020** | **AAPC (95% CI) ^†^** |  | **Year** | **APC (95% CI) ^‡^** |  | **Year** | **APC (95% CI) ^‡^** |
| **Ischemic Heart Disease** | | | | | | | | | |  |
|  | 18 - 44 years | 555.9 | 715.0 | 2.7 (-1.8, 7.4) |  | 2014 - 2020 | 2.7 (-1.8, 7.4) |  |  |  |
|  | 45 - 64 years | 1575.3 | 2127.2 | 4.9 (3.0, 6.9)* |  | 2014 - 2018 | 1.5 (-7.9, 11.7) |  | 2018 - 2020 | 6.7 (2.8, 10.7)* |
|  | 65 - 74 years | 2205.6 | 2722.8 | 3.4 (2.0, 4.9)* |  | 2014 - 2020 | 3.4 (2.0, 4.9)*** |  |  |  |
|  | ≥ 75 years | 3108.0 | 3529.8 | 2.2 (1.6, 2.8)* |  | 2014 - 2020 | 2.2 (1.6, 2.8)*** |  |  |  |
| **Acute myocardial infraction** | | | | | | | | | |  |
|  | 18 - 44 years | 61.5 | 37.2 | -7.3 (-12.5, -1.8)* |  | 2014 - 2020 | -7.3 (-12.5, -1.8)* |  |  |  |
|  | 45 - 64 years | 114.0 | 112.4 | 0.0 (-1.2, 1.2) |  | 2014 - 2020 | 0.0 (-1.2, 1.2) |  |  |  |
|  | 65 - 74 years | 155.0 | 138.0 | -1.3 (-4.5, 1.9) |  | 2014 - 2020 | -1.3 (-4.5, 1.9) |  |  |  |
|  | ≥ 75 years | 294.3 | 272.9 | -1.0 (-3.3, 1.4) |  | 2014 - 2020 | -1.0 (-3.3, 1.4) |  |  |  |
| **Peripheral Arterial Disease** | | | | | | | | | |  |
|  | 18 - 44 years | 111.7 | 137.8 | 3.2 (-2.6, 9.4) |  | 2014 - 2017 | 15.9 (-4.6, 40.7) |  | 2017 - 2020 | -8.0 (-22.1, 8.6) |
|  | 45 - 64 years | 246.4 | 335.6 | 4.7 (2.2, 7.4)* |  | 2014 - 2018 | 7.5 (2.3, 13.0)* |  | 2018 - 2020 | -0.6 (-12.9, 13.4) |
|  | 65 - 74 years | 291.4 | 444.9 | 6.6 (4.4, 8.9)* |  | 2014 - 2020 | 6.6 (4.4, 8.9)** |  |  |  |
|  | ≥ 75 years | 390.0 | 614.2 | 7.9 (4.7, 11.1)* |  | 2014 - 2018 | 10.5 (3.8, 17.6)* |  | 2018 - 2020 | 2.8 (-11.3, 19.3) |
| **Major LEA** | | | | | | | | | |  |
|  | 18 - 44 years | 14.0 | 7.4 | -12.8 (-24.2, 0.4) |  | 2014 - 2020 | -12.8 (-24.2, 0.4) |  |  |  |
|  | 45 - 64 years | 20.0 | 16.2 | -5.7 (-10.7, -0.3)* |  | 2014 - 2020 | -5.7 (-10.7, -0.3)* |  |  |  |
|  | 65 - 74 years | 16.6 | 15.5 | -1.1 (-6.8, 5.0) |  | 2014 - 2020 | -1.1 (-6.8, 5.0) |  |  |  |
|  | ≥ 75 years | 15.8 | 15.2 | -5.0 (-11.8, 2.5) |  | 2014 - 2020 | -5.0 (-11.8, 2.5) |  |  |  |
| **Minor LEA** | | | | | | | | | |  |
|  | 18 - 44 years | 53.1 | 22.3 | -14.6 (-21.8, -6.7)* |  | 2014 - 2020 | -14.6 (-21.8, -6.7)** |  |  |  |
|  | 45 - 64 years | 42.1 | 31.8 | -3.2 (-6.9, 0.6) |  | 2014 - 2020 | -3.2 (-6.9, 0.6) |  |  |  |
|  | 65 - 74 years | 32.7 | 28.4 | -0.5 (-6.3, 5.6) |  | 2014 - 2020 | -0.5 (-6.3, 5.6) |  |  |  |
|  | ≥ 75 years | 29.0 | 20.4 | -3.7 (-8.2, 1.0) |  | 2014 - 2020 | -3.7 (-8.2, 1.0) |  |  |  |
| **Diabetic foot and peripheral angiopathy** | | | | | | | | | |  |
|  | 18 - 44 years | 142.5 | 162.0 | 1.7 (-3.5, 7.1) |  | 2014 - 2020 | 1.7 (-3.5, 7.1) |  |  |  |
|  | 45 - 64 years | 232.0 | 241.6 | 0.9 (-1.3, 3.3) |  | 2014 - 2020 | 0.9 (-1.3, 3.3) |  |  |  |
|  | 65 - 74 years | 238.8 | 259.3 | 1.2 (-1.3, 3.8) |  | 2014 - 2020 | 1.2 (-1.3, 3.8) |  |  |  |
|  | ≥ 75 years | 311.6 | 334.7 | 1.9 (0.1, 3.8)* |  | 2014 - 2020 | 1.9 (0.1, 3.8)* |  |  |  |
| **Stroke** | | | | | | | | | |  |
|  | 18 - 44 years | 203.9 | 292.3 | 6.6 (2.7, 10.7)* |  | 2014 - 2020 | 6.6 (2.7, 10.7)** |  |  |  |
|  | 45 - 64 years | 499.9 | 748.9 | 7.3 (4.8, 9.8)* |  | 2014 - 2016 | 12.7 (-1.7, 29.3) |  | 2016 -2020 | 4.7 (1.2, 8.3)** |
|  | 65 - 74 years | 803.7 | 1065.8 | 4.9 (3.9, 5.8)* |  | 2014 - 2020 | 4.9 (3.9, 5.8)*** |  |  |  |
|  | ≥ 75 years | 1284.4 | 1629.8 | 3.8 (2.7, 5.0)* |  | 2014 - 2020 | 3.8 (2.7, 5.0)*** |  |  |  |
| **Diabetic eye complications** | | | | | | | | | |  |
|  | 18 - 44 years | 349.2 | 1035.2 | 17.9 (2.9, 35.1)* |  | 2014 - 2020 | 17.9 (2.9, 35.1)* |  |  |  |
|  | 45 - 64 years | 431.1 | 1283.8 | 19 (7.3, 31.8)* |  | 2014 - 2020 | 19 (7.3, 31.8)** |  |  |  |
|  | 65 - 74 years | 395.4 | 1296.7 | 19.2 (5.7, 34.3)* |  | 2014 - 2020 | 19.2 (5.7, 34.3)* |  |  |  |
|  | ≥ 75 years | 464.8 | 1137.9 | 16.4 (6.4, 27.4)* |  | 2014 - 2020 | 16.4 (6.4, 27.4)** |  |  |  |
| **Nephropathy** | | | | | | | | | |  |
|  | 18 - 44 years | 1427.4 | 2822.6 | 13.9 (6.1, 22.2)* |  | 2014 - 2020 | 13.9 (6.1, 22.2)** |  |  |  |
|  | 45 - 64 years | 1950.5 | 3658.5 | 13.6 (5.9, 21.7)* |  | 2014 - 2020 | 13.6 (5.9, 21.7)** |  |  |  |
|  | 65 - 74 years | 3150.3 | 4880.6 | 9.6 (4.5, 14.9)* |  | 2014 - 2020 | 9.6 (4.5, 14.9)** |  |  |  |
|  | ≥ 75 years | 4840.9 | 6495.8 | 6.5 (2.9, 10.3)* |  | 2014 - 2020 | 6.5 (2.9, 10.3)** |  |  |  |
| **Neuropathy** | | | | | | | | | |  |
|  | 18 - 44 years | 128.5 | 156.4 | 3.2 (-2.3, 9.0) |  | 2014 - 2020 | 3.2 (-2.3, 9.0) |  |  |  |
|  | 45 - 64 years | 136.4 | 210.8 | 9.3 (5.1, 13.6)* |  | 2014 - 2020 | 9.3 (5.1, 13.6)** |  |  |  |
|  | 65 - 74 years | 111.9 | 224.1 | 11.6 (7.7, 15.5)* |  | 2014 - 2020 | 11.6 (7.7, 15.5)*** |  |  |  |
|  | ≥ 75 years | 168.5 | 300.9 | 9.8 (6.8, 12.9)* |  | 2014 - 2020 | 9.8 (6.8, 12.9)*** |  |  |  |

^†^ Joinpoint regression software provided indication whether AAPC p-value is significant at p<0.05, no detailed p-values were provided.

^‡^ Joinpoint regression software provides detailed p-values for APC.

* P < 0.05

** P < 0.01

*** P < 0.005
